# Unresolved Issues in Familial Mediterranean Fever: Is p.R202Q *MEFV* Variant Potentially Pathogenetic in Unleashing Inflammation?

**DOI:** 10.1007/s10875-025-01898-8

**Published:** 2025-06-10

**Authors:** Chiara Baggio, Francesca Oliviero, Paola Galozzi, Irina Guidea, Andrea Doria, Roberta Ramonda, Sara Bindoli, Paolo Sfriso

**Affiliations:** 1 Department of Medicine, DIMED, University of Padova, Padova, Italy; 2 Rheumatology Unit, Hospital of Padova, Padova, Italy; 3 Laboratory Medicine Unit, Hospital of Padova, Padova, Italy

**Keywords:** Pyrin, Inflammasome, Inflammation, Cytology, Neutrophils, Cytokines

## Abstract

Familial Mediterranean Fever (FMF) is caused by mutations in the *MEFV* gene, which encodes for pyrin. Although genetic testing is commonly employed for FMF diagnosis, the interpretation of genetic results is often challenging. Therefore, we aimed to functionally characterise the p.R202Q *MEFV* alteration. Furthermore, we hypothesized that inflammation may affect genomic stability and neutrophilic (N) subsets. A cohort comprising patients with FMF (*n* = 4), p.R202Q variant (*n* = 18) and FMF-like (*n* = 8) were selected from the Outpatient Clinic for Autoinflammatory diseases of Padova University Hospital. Primary monocytes were incubated for 3 h in the presence of LPS or LPS + PKN1/2 inhibitor (UCN-01). Colchicine pretreatment was applied to assess its anti-inflammatory effect. Pro-inflammatory cytokines were measured by ELISA and leukocytes were examined using May-Grünwald-Giemsa staining on blood smears. We did not find significant differences in IL-1 and IL-18 levels in monocytes treated with LPS + UCN-01 between p.R202Q patients and healthy donors (HDs). The levels of IL-1β released from LPS-stimulated patients were higher in p.R202Q patients than in HDs. We found that immature and hypersegmented neutrophils were higher in p.R202Q patients than in HD. Nuclear abnormalities were higher in FMF and p.R202Q patients than in HD. Finally, we found a higher cell rate in leukocytes from p.R202Q patients than in HDs. The p.R202Q variant did not appear to affect pyrin function, albeit these patients presented cytological alterations similar to those observed in FMF patients. These changes may contribute to FMF pathophysiology by influencing inflammation progression.

**Supplementary Information:**

The online version contains supplementary material available at 10.1007/s10875-025-01898-8.

## Introduction

Familial Mediterranean Fever (FMF) is the most frequent hereditary autoinflammatory disorder and is characterized by recurrent attacks of fever and serositis, leading to severe chest, abdominal, or joint pain [1, 2].

Colchicine decreases chronic inflammation and it is the gold standard treatment for FMF with a non-responder rate of only 10% [3, 4]. For patients who are intolerant or resistant to colchicine, interleukin (IL)-1 inhibitors are effective in reducing disease flares [5].

The diagnosis of FMF relies on clinical criteria, further supported by review of ethnic origin, family history, response to colchicine therapy, and genetic test [4, 6].

FMF is associated with mutations in the *MEFV* gene which encodes the pyrin protein, an important inflammasome regulator that detects the Rho-GTPase activity. Therefore, in FMF patients an uncontrollable inflammatory response occurs due to excessive secretion of IL-1β [7].

Although genetic screening confirms the diagnosis upon identification of mutations on *MEFV*, a large part of the reported variants are categorized as “variants of unknown significance” (VUS) whose diagnostic value remains unclear [6, 8]. Furthermore, FMF has an autosomal recessive pattern of inheritance that can sometimes be inherited as an autosomal dominant trait, and symptomatic patients with heterozygous mutations have been reported [9–12].

Functional assays are necessary to sustain the diagnosis and discriminate pathogenic variants from non-pathogenic *MEFV* polymorphisms. In fact, Van Gorp et al. and Magnotti F. developed a functional assay that robustly discriminates pathogenic *MEFV* mutations from polymorphisms [4, 6]. The functional assay is based on the activation of pyrin regulated by two independent control mechanisms. Normally, pyrin is maintained inactive by two kinases (PKN1/2) that mediate pyrin phosphorylation in two serine residues (S208 and S242) (first control mechanism) and its sequestration by 14-3‐3 chaperone proteins [13, 14]. In healthy donors (HDs), pyrin inflammasome activation appears to depend on microtubule dynamics, which have been proposed as a second control mechanism [14]. PKC inhibitors trigger inflammasome activation only in FMF monocytes, which are believed to lack this secondary regulatory mechanism [6].

Although the p.R202Q (c.605G > A) variant in exon 2 is commonly described as a polymorphism (GnomAD total allele frequency 23%), recent reports have shown that it may be a disease-causing mutation [1, 15]. It is reported that the p.R202Q variant may cause FMF symptoms depending on whether it is homozygous or compound heterozygous and requires colchicine treatment [15, 16]. Moreover, a study by Ritis et al., reported no p.R202Q homozygous mutation in healthy controls, suggesting that the p.R202Q variation may be a mutation rather than a polymorphism [17, 18].

We detected p.R202Q *MEFV* variant in 18 patients with suspected FMF/FMF-like condition.

Therefore, our aim was to investigate the clinical significance of *MEFV* p.R202Q alteration through a functional characterization in vitro of this variant. In addition, we performed a cytologic evaluation of leukocytes from FMF and p.R202Q patients to investigate the phenotypical characteristics of neutrophils (N), the most abundant circulating leukocytes during inflammatory attacks in FMF, and to detect the presence of genomic instability.

## Methods

### Subjects

Four patients with genetically confirmed FMF, along with eighteen with p.R202Q *MEFV* variation and eight patients with FMF-like with K695R, E148Q or E195D variations were chosen from the outpatient clinic for Autoinflammatory diseases. Sanger sequencing was employed to assess the carriage of the variations in the *MEFV*,* MVK* and *TNFRSF1A* gene. The carriers of the p.R202Q variant (NM_000243.3) were selected upon the presence of recurrent fever and clinical manifestations as requested by TelHashomer Criteria, while the potential carriage of *MEFV* variations was not assessed in HDs. Demographical data, clinical features, and genetics of patients are depicted in Table 1. All FMF patients fulfilled the Tel HaShomer criteria for FMF and had at least one mutation in the *MEFV* gene. Blood samples were collected during the follow-up visit. Eighteen HDs were included in the study and were not affected by relevant comorbidities. Blood samples from HDs were drawn on the same day as patients. All subjects gave their fully informed written consent to participate in the study, which was carried out in compliance with the principles of the Declaration of Helsinki. The protocol follows the guidelines of the Ethics Committee of Padova University Hospital (protocol code 5349/AO/22).

**Table 1:** Demographical data, clinical features and genetic of FMF, FMF-like and p.R202Q patients

	FMF	FMF-like	*p*.R202Q
**Patients**,** n**	4	8	18
**Sex**,** n (%)**	F: 2 (50), M: 2 (50)	F: 5 (63), M: 3 (37)	F: 12 (67), M: 6 (33)
**Age**,** years (IQR)**	30 (22–35)	34 (22–47)	33 (25–50)
**Age of onset**,** years (IQR)**	17 (15–22)	18 (15–34)	18 (15–47)
**Characteristics of febrile episodes**			
**Fever**,** n (%)** *no fever*	4 (100) 0 (0)	7 (87.5) 1 (12.5)	16 (88.9) 2 (11.1)
*< 38 °C* *= 38 °C* *> 38 °C*	0 (0) 0 (0) 4 (100)	0 (0) 5 (62.5) 2 (25)	2 (11.1) 6 (33.3) 8 (44.5)
**Symptoms**			
**Patients with serositis** Peritonitis Pericarditis	3 (75) 3 (75) 0 (0)	5 (62.5) 5 (62.5) 3 (37.5)	8 (44.4) 5 (27.7) 5 (27.7)
**Arthritis** **Arthralgias** **Skin manifestations** **Pharyngodynia** **Oral aphthosis** **Headache** **Asthenia**	0 (0) 2(50) 1 (25) 0 (0) 1 (25) 0 (0) 1 (25)	0 (0) 4 (50) 2 (25) 1 (12.5) 5 (62.5) 1 (12.5) 1 (12.5)	1 (5.6) 9 (50) 5 (27.7) 3 (16.7) 4 (22.2) 2 (11.1) 8 (44.4)
**Genotype**			
**Genotype**,** n (%)** *MEFV* *IFIH1* *TNFRSF1A* *MVK*	p.E148Q/p.M680I het: 2 (50) p.M680I hom: 1 (25) p.M694I/p.V726A het: 1 (25)	p.E195D het: 1 (12.5) p.K695R het: 4 (50) p.E148Q het: 2 (25) p.E148Q/p.P369S/p.R408Q het: 1 (12.5)	p.R202Q het: 11 (61.1) p.R202Q hom: 7 (38.9) p.R186C het: 1 (5.5) p.R92Q het: 1 (5.5) p.V5F het: 1 (5.5)
**Pharmacological treatment**			
**Colchicine**,** n (%)**	4 (100)	5 (62.5)	15 (83.3)
Ineffectiveness, n (%) Partial response, n (%) Complete response, n (%) Intolerance, n (%)	0 (0) 1 (25) 3 (75) 0 (0)	2 (25) 1 (12.5) 4 (50) 1 (12.5)	2 (11.1) 2 (11.1) 12 (66.7) 1 (5.69
**Anakinra**,** n (%)**	1 (25)	0 (0)	1 (5.6)
**Canakinumab**,** n (%)**	0 (0)	3 (37.5)	2 (11.1)
**None**,** n (%)**	0 (0)	1 (12.5)	2 (11.1)

Data are expressed as the median and interquartile range (IQR). p calculated according Kruskal–Wallis test. Dunn’s post hoc test

### Monocyte Isolation

Blood was drawn in heparin-coated tubes from FMF (*n* = 3), FMF-like (*n* = 5), p.R202Q (*n* = 15) patients, and HDs (*n* = 12). Peripheral blood mononuclear cells (PBMCs) were isolated by density-gradient centrifugation using Histopaque 1077 (Sigma-Aldrich, St. Louis, Missouri, USA). PBMCs were seeded into 96-well plates at 2.5 × 10^5^ cells/well, in RPMI 1640 (Sigma-Aldrich) supplemented with 10% fetal calf serum (FBS) with 1% glutamine (Sigma-Aldrich) and 1% penicillin-streptomycin (Sigma-Aldrich). Monocytes were isolated from mononuclear cells by exploiting their ability to adhere to plastic. The next day, the supernatant was removed and replaced with complete medium.

### Functional Assay

Primary monocytes were incubated for 3 h in the presence of LPS (10 ng/ml, InvivoGen, Toulouse, France). After LPS stimulation, monocytes were treated with UCN-01 (12.5 µM, Sigma-Aldrich) for 1.5 h. When indicated, monocytes were also pretreated with colchicine (1 µM, Sigma-Aldrich) for 30 min before the use of UCN-01 to assess its anti-inflammatory effect. The culture supernatants and cell lysates were collected for subsequent cytokine determination. The same procedure was performed on the whole blood of selected patients (FMF *n* = 3, p.R202Q *n* = 3, HDs *n* = 7). Whole blood was diluted 1:2 with PBS and incubated at 37 °C for 1 h with LPS (10 ng/mL) and then for 1.5 h with UCN-01 (12.5 µM). Finally, we collected plasma by centrifugation for subsequent cytokine detection (400 g for 10 min).

### Cytokine Detection

The following cytokines were measured in plasma from HDs, FMF and p.R202Q patients and in monocyte supernatant and lysate using commercially available enzyme-linked immunosorbent assay (ELISA) kits: interleukin (IL)-1β (sensitivity: 0.5 pg/mL; BioLegend, San Diego California, USA), IL-α (sensitivity: 0.6 pg/mL; BioLegend), IL-18 (sensitivity: 5.5 pg/mL; R&D Systems, Minneapolis, USA).

### Cellular Morphology and Cytogenic Evaluation of Leukocytes

May-Grünwald-Giemsa (MGG) staining was used for studying cellular morphology and to perform a cytogenic evaluation of leucocytes. Blood samples from FMF (*n* = 4), FMF-like (*n* = 8), p.R202Q patients (*n* = 18) and HDs (*n* = 18) were collected in EDTA tubes. Air-dried blood smears were stained using a stepwise procedure with MGG (Sigma-Aldrich) and oil immersion microscopy with 1000× magnification was applied for the cytological analysis as previously described [19]. Results were finally expressed as a percentage of total leukocytes counted in a slide. All slides were examined for the presence of vacuoles and different types of nuclear abnormalities (NA) including micronuclei (MNi) and buds, binucleated cells (the presence of two similar nuclei in a cell), hypersegmented N, and cell death [19, 20].

We calculated the Immature N/ total N ratio (I/T) (left shift) by dividing the percentage of immature N (bands) by the percentage of total N. The study group was classified according to the I/T ratio into normal (< 0.2), moderate shift to the left (0.2–0.29) and severe shift to the left (≥ 0.3) [21].

### Statistic Analysis

The Shapiro-Wilk test was used to analyse the distribution of continuous variables, and variables with a non-normal distribution were presented as medians with the corresponding interquartile range (IQR). As no variable was normally distributed among the categories, only non-parametric tests were used. Kruskal Wallis followed by Dunnet post hoc tests were used for multiple comparisons. The differences between the two groups of patients were tested using the Mann-Whitney U test. Spearman correlation analysis was used to determine the correlations. Statistical analysis was performed with GraphPad Prism 8 (GraphPad Software Inc., La Jolla, CA, USA). A p-value < 0.05 was considered significant.

## Results

### Demographical Data, Clinical Features, Genetic and Pharmacological Treatment of FMF, FMF-like and p.R202Q Carrier Patients

Demographical data, clinical features, genetic and pharmacological treatment of all patients included in the study are depicted in Table 1. Most of the patients’ samples were collected out of the disease attacks, or during intercritical phases, therefore inflammatory biomarkers such as C-reactive protein (CRP) and serum amyloid A (SAA) were mostly in the range of normality (Table S1). No differences regarding the age of symptoms’ onset were observed between FMF, FMF-like and p.R202Q carrier patients. It worth noting that most of our patients were diagnosed during adulthood, and only 8 (27%) of the entire cohort presented an early onset during infancy; indeed, the mean age of symptom’s onset was during adolescence, between 16 and 19 years. Patients carrying the p.R202Q variation were diagnosed as FMF since fulfilling the Tel Hashomer criteria. Almost all p.R202Q carriers had recurrent episodes of fever lasting 1–2 days. In addition, as reported in Tables 1 and 44% had associated serositis, and 50% presented with arthralgia. Serositis was instead present in 75% of FMF, however no differences about the overall serositis frequency were observed between FMF-like (63%) and p.R202Q (44%). Chest pain was equally represented amongst the three groups, while abdominal pain predominated in FMF (75%). Skin manifestation was equally represented amongst the three groups, while pure arthritis was rarely described.

No difference in the colchicine treatment response was observed between the different groups examined. Overall, 67% of the p.R202Q carriers responded to colchicine treatment, while 11% did not. No significant difference was detected between colchicine-responders and non-responders concerning genotyping.

### Plasma Cytokines Levels in FMF, FMF-like, p.R202Q Carrier Patients and HDs

**Fig. 1:**
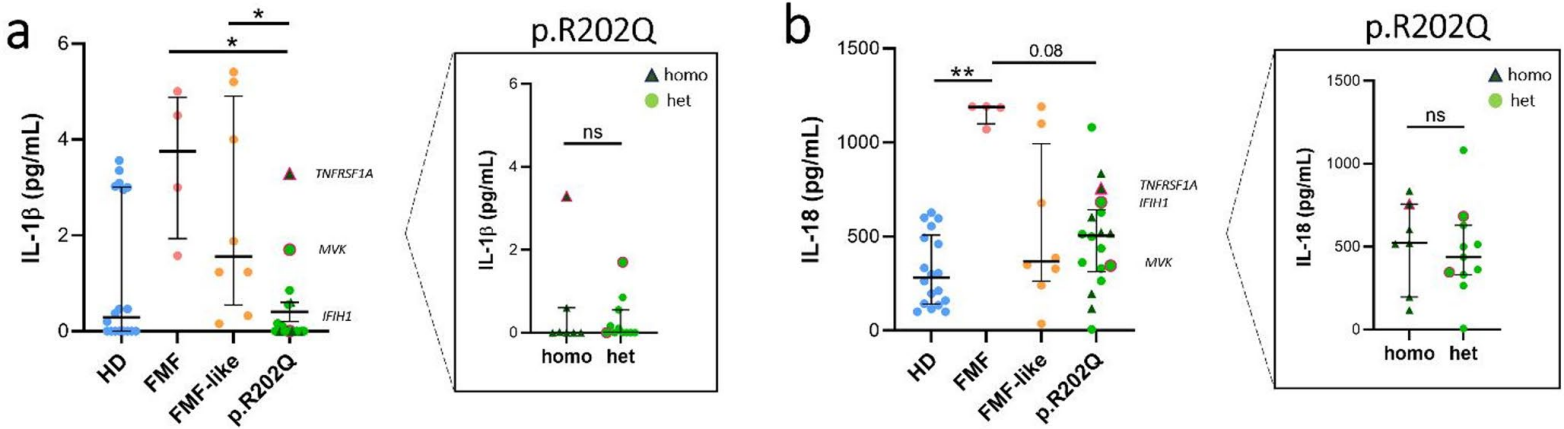
Plasma cytokines levels in FMF, FMF-like, p.R202Q patients and HD. Plasma cytokines levels in HD (*n* = 18), FMF (*n* = 4), FMF-like (*n* = 8) and p.R202Q patients (*n* = 18) were evaluated by ELISA as described in Materials and Methods. p.R202Q patients were divided into homozygous (*n* = 7) and heterozygous (*n* = 11) groups for comparison. **(a)** IL-1β levels. **(b)** IL-18 levels. Data are expressed as the median and IQR. p calculated according to Kruskal Wallis test. Dunn’s post hoc test: *p < 0.05, **p < 0.01. The difference between homozygous vs. heterozygous groups was evaluated using the Mann Whitney test, ns. Abbreviations are as follows: FMF, Familial Mediterranean Fever; HD, healthy donors; homo, homozygous; het, heterozygous

IL-1β plasma levels were higher in FMF and FMF-like than p.R202Q patients (Fig. 1a). IL-18 levels were higher in FMF patients vs. other groups analyzed, although the difference achieved a statistically significant difference only in the comparison with HDs (Fig. 1b). We found no significant differences between the IL-1β and IL-18 plasma levels of p.R202Q patients and HDs. IL-1α levels were below the detection limit of the ELISA kit in all groups. Statistical analysis showed no significant differences in plasma IL-1β and IL-18 levels between patients homozygous and heterozygous for the p.R202Q variation.

### Inflammasome Activation in Monocytes from FMF, FMF-like, p.R202Q Carrier Patients: IL-1β and IL-18 Evaluation

**Fig. 2:**
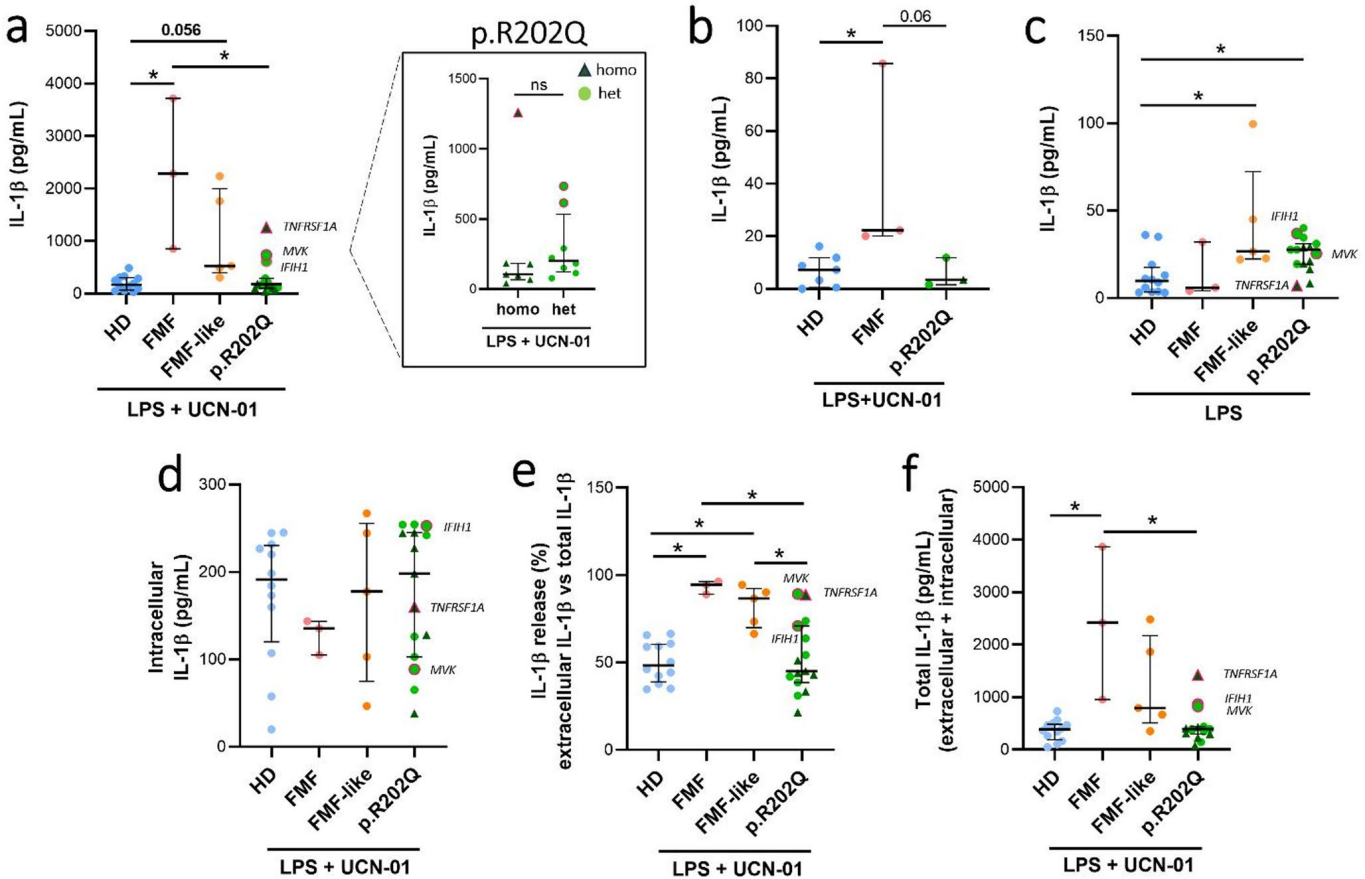
Evaluation of IL-1β levels in monocytes and whole blood from FMF, FMF-like and p.R202Q carrier patients following LPS + UCN-01 treatment. Monocytes from HD (*n* = 12), FMF patients (*n* = 3), FMF-like patients (*n* = 5) and p.R202Q patients (*n* = 15) and whole blood from HD (*n* = 7), FMF patients (*n* = 3) and p.R202Q patients (*n* = 3) were treated with 12.5 µM UCN-01 after LPS priming (10 ng/mL) as described in Materials and Methods. p.R202Q patients were divided into homozygous (*n* = 7) and heterozygous (*n* = 8) groups for comparison. IL-1β levels were quantified by ELISA.**(a)** Extracellular IL-1β levels in monocytes after LPS + UCN-01 treatment.**(b)** IL-1βlevels after whole blood stimulation with LPS + UCN-01.**(c)**Extracellular IL-1β levels in monocytes after LPS stimulation. **(d)**Intracellular IL-1β levels (pro IL-1 β).**(e)**% of IL-1β release.**(f)** Total IL-1β levels (extracellular + intracellular IL-1β). Data are shown as the median (IQR). p calculated according to the Kruskal-Wallis test. Dunn’s post hoc test: *p < 0.05. The difference between homozygous vs. heterozygous groups was evaluated using the Mann Whitney test, ns. Abbreviations are as follows: FMF, Familial Mediterranean Fever; HD, healthy donors; homo, homozygous; het, heterozygous

**Fig. 3:**
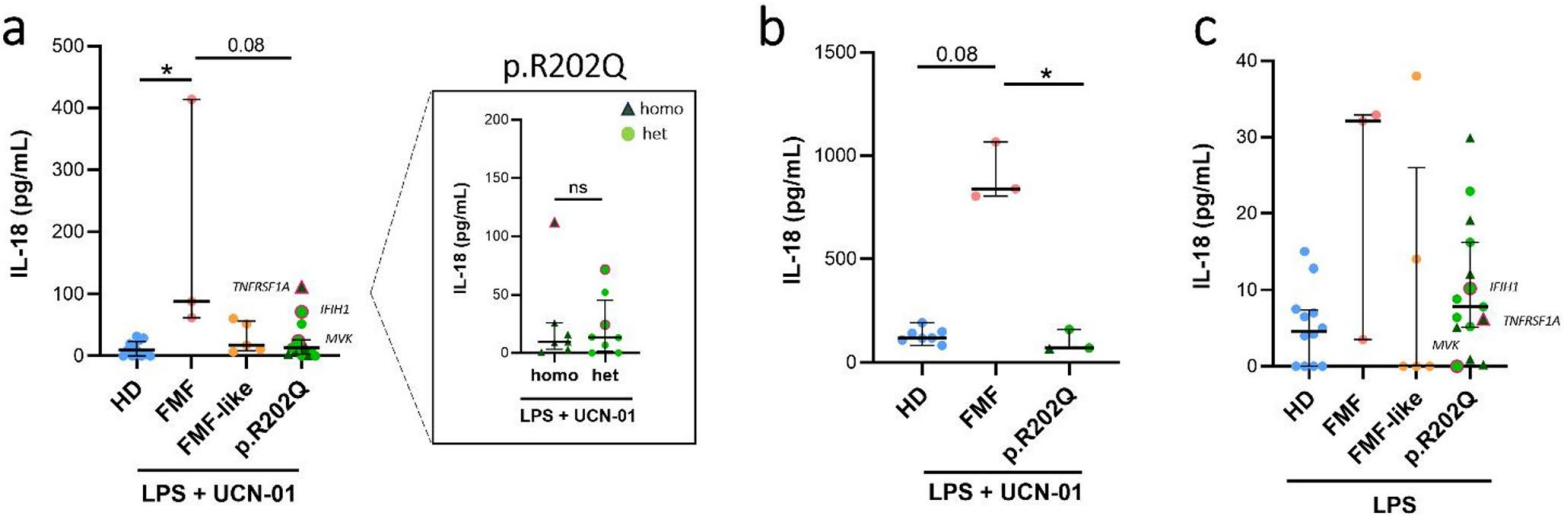
Evaluation of IL-18 levels in monocytes and whole blood from FMF, FMF-like and p.R202Q carrier patients following LPS + UCN-01 treatment. Monocytes from HD (*n* = 12), FMF patients (*n* = 3), FMF-like patients (*n* = 5) and p.R202Q patients (*n* = 15) and whole blood from HD (*n* = 7), FMF patients (*n* = 3) and p.R202Q patients (*n* = 3) were treated with 12.5 µM UCN-01 after LPS priming (10 ng/mL) as described in Materials and Methods. p.R202Q patients were divided into homozygous (*n* = 7) and heterozygous (*n* = 8) groups for comparison. IL-18 levels were quantified by ELISA.**(a)** IL-18 levels in monocytes after LPS + UCN-01 treatment.**(b)** IL-18 levels after whole blood stimulation with LPS + UCN-01.**(c)** IL-18 levels in monocytes after LPS stimulation. Data are shown as the median (IQR). p calculated according to the Kruskal-Wallis test. Dunn’s post hoc test: *p < 0.05. The difference between homozygous vs. heterozygous groups was evaluated using the Mann Whitney test, ns. Abbreviations are as follows: FMF, Familial Mediterranean Fever; HD, healthy donors; homo, homozygous; het, heterozygous

To explore the mechanisms underlying deregulation of the pyrin inflammasome in p.R202Q patients, we assessed the efficacy of UCN-01 to trigger IL-1β and IL-18 release in primary monocytes from HDs (*n* = 12), FMF (*n* = 3), FMF-like (*n* = 5) and p.R202Q (*n* = 15) patients (Figs. 2 and 3, Figure S1a). After LPS + UCN-01 treatment, we found no significant differences between the release of IL-1β from monocytes of p.R202Q and HDs (Fig. 2a). In our experimental conditions, monocytes from 12 HDs (100%) and 12 p.R202Q patients (80%) released < 500 pg mL of IL-1β. In sharp contrast, monocytes from FMF patients released higher levels of IL-1β than HDs and p.R202Q patients, leading to an average level of 12-fold higher than the average level in the supernatant of HDs monocytes and of 8-fold higher than the average level of p.R202Q monocytes. Although not significant (*p* = 0.056), IL-1β levels released from FMF-like monocytes were higher than in monocytes from HDs. No differences were observed in IL-1β release from monocytes between homozygous (homo) and heterozygous (het) p.R202Q patients after LPS + UCN-01 treatment (Fig. 2a). Preliminary experiments on a small number of patients (FMF *n* = 3, p.R202Q *n* = 3) and HDs (*n* = 7) were also conducted on whole blood (Fig. 2b). The results were comparable to those obtained with the functional assay conducted on monocytes. No differences were observed in IL-1β levels between HDs and p.R202Q patients after LPS + UCN-01 treatment. Higher levels of IL-1β were found in FMF patients than HDs and p.R202Q patients. An interesting difference in IL-1β release emerged between p.R202Q patients and healthy donors following LPS stimulation of monocytes (Fig. 2c). In monocytes treated with LPS + UCN-01, intracellular IL-1β (pro IL-1β) levels were lower in FMF patients than other groups (Fig. 2d). Although this value was not significant, it could indicate a higher release of IL-1β following treatment with UCN-01 due to pyrin activation. Indeed, in monocytes treated with LPS + UCN-01, the levels of extracellular IL-1β compared to the total levels of IL-1β (extracellular + intracellular IL-1β) were higher in FMF patients compared to HDs and p.R202Q patients (Fig. 2e). In addition, IL-1β was also higher in FMF-like patients than in HDs and p.R202Q patients. Finally, total IL-1β levels were higher in FMF and in FMF-like patients than in p.R202Q patients and HDs (Fig. 2f).

We evaluated IL-18 release from monocytes stimulated with LPS and subsequently treated with UCN-01 in FMF, FMF-like, p.R202Q patients, and HDs (Fig. 3a, Figure S1b). We found no significant differences between the IL-18 levels released from p.R202Q monocytes and HDs. Monocytes from FMF patients released higher levels of IL-18 than monocytes from HDs and, although not significant (*p* = 0.08), IL-18 levels released from FMF monocytes were higher than in monocytes from p.R202Q and FMF-like patients (Fig. 3a). After LPS + UCN-01 treatment, no differences were observed in IL-18 release from monocytes between homo and het p.R202Q patients (Fig. 3a).

No differences were observed in IL-18 levels between HDs and p.R202Q patients after whole blood stimulation with LPS + UCN-01. Instead, higher levels of IL-18 were found in FMF patients than HDs and p.R202Q patients (Fig. 3b). Finally, no differences in IL-18 release emerged between p.R202Q patients and healthy donors following LPS stimulation of monocytes (Fig. 3c).

Overall, these results indicate strongly differing inflammasome responses to UCN-01 between FMF and p.R202Q patients.

### Colchicine Treatment Blocks Inflammasome Activation only in FMF Patients

**Fig. 4:**
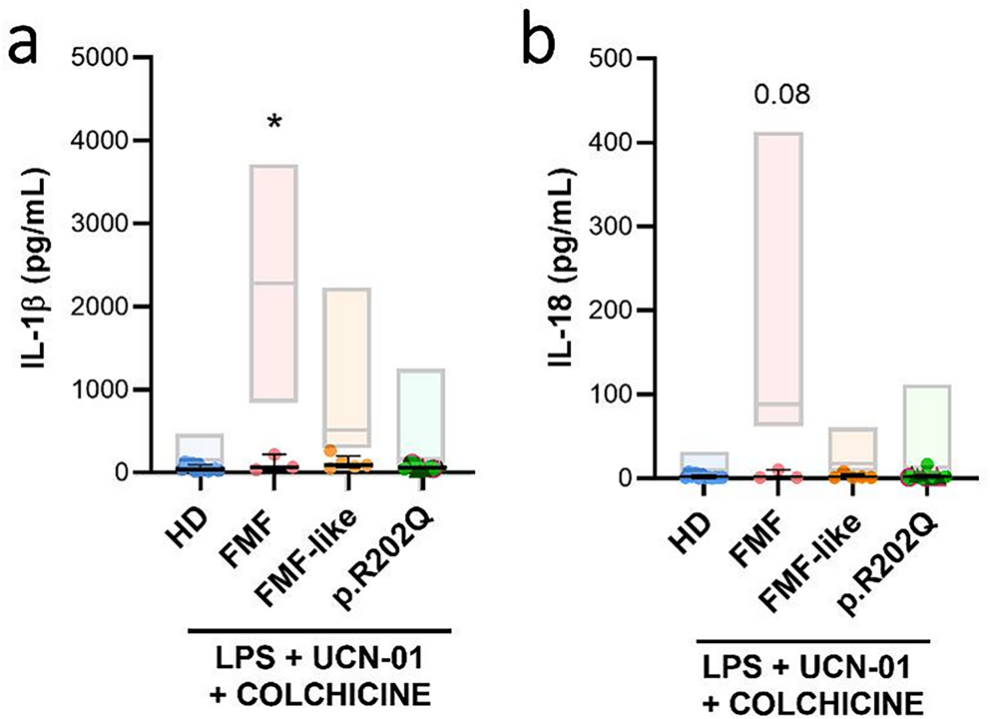
Colchicine blocked inflammasome activation only in monocytes from FMF patients following LPS + UCN-01 treatment. Monocytes from HD (*n* = 12), FMF patients (*n* = 3), FMF-like patients (*n* = 5) and p.R202Q patients (*n* = 15) were treated with 12.5 µM UCN-01 after LPS priming (10 ng/mL). Colchicine (1 µM) was added 30 min before addition of 12.5 µM UCN-01 as described in Materials and Methods. IL-1β and IL-18 levels were quantified by ELISA.**(a)** IL-1β levels after colchicine treatment.**(b)** IL-18 levels after colchicine treatment. Data are shown as the median (IQR). p calculated according to the Mann Whitney test: *p < 0.05. Abbreviations are as follows: FMF, Familial Mediterranean Fever; HD, healthy donors

To assess the effect of colchicine on inflammasome activation mediated by UCN-01, we measured IL-1β and IL-18 levels in culture supernatants of LPS-primed monocytes stimulated with UCN-01, with or without colchicine. In FMF patients, colchicine treatment led to a marked reduction in cytokine release, with IL-1β levels decreasing from a median of 2285 pg/ml to 66.0 pg/ml (Fig. 4a) and IL-18 from 1188 pg/ml to 1.0 pg/ml (Fig. 4b). In contrast, in monocytes from HDs and p.R202Q carriers, cytokine levels remained low following UCN-01 stimulation, and colchicine treatment did not result in a significant additional decrease. These findings indicate that colchicine selectively inhibits pyrin inflammasome activation in FMF monocytes, where a pathogenic *MEFV* variant drives a heightened inflammatory response. In p.R202Q patients, colchicine reduced the release of IL-18 and IL-1β only in the three individuals who exhibited inflammasome activation following LPS + UCN-01 treatment and who also carried mutations in the *IFIH1*, *MVK*, or *TNFRSF1A* genes (Figure S2). As UCN-01 does not robustly activate the pyrin inflammasome in non-FMF individuals, colchicine’s effect in these groups is not biologically meaningful.

### IL-1α Release after UCN-01 Treatment from Monocytes in FMF, FMF-like and p.R202Q Carrier Patients

**Fig. 5:**
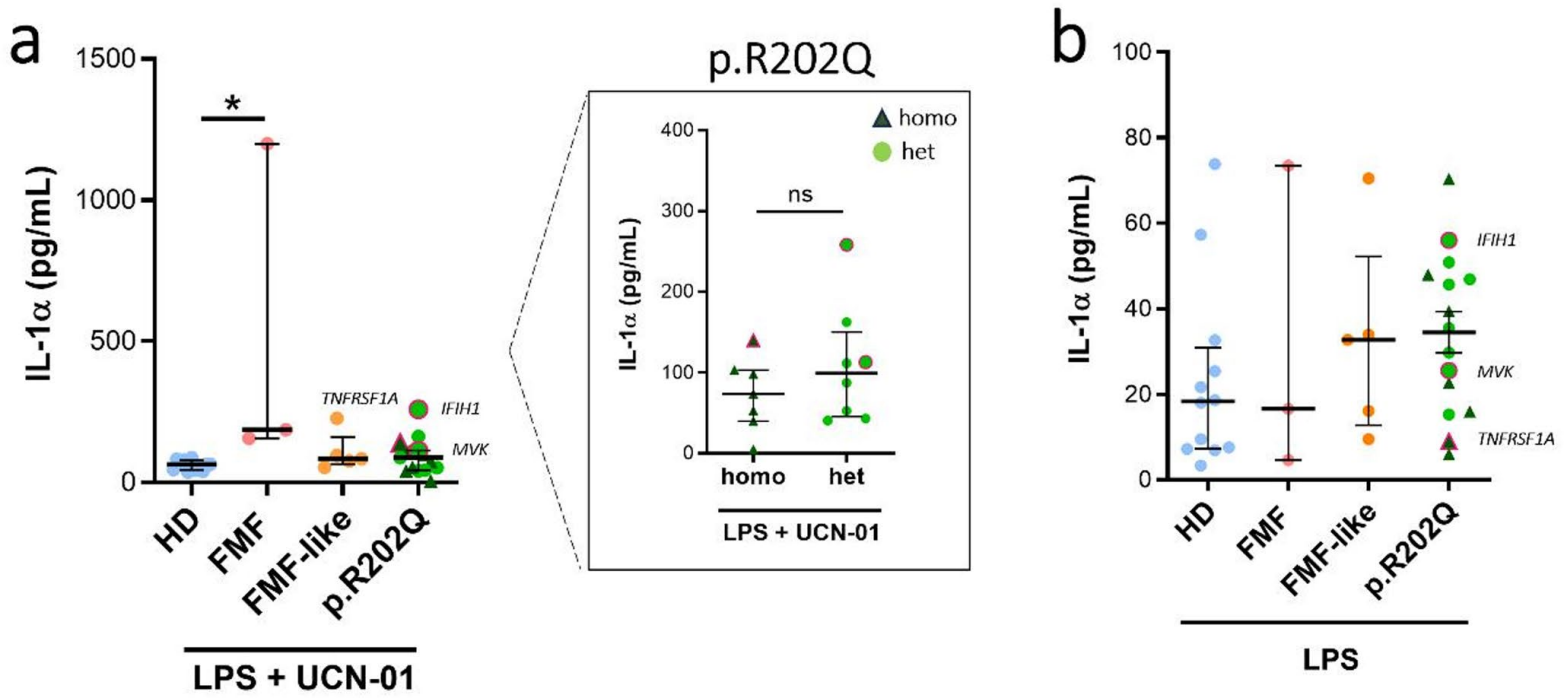
IL-1α release in monocytes from FMF, FMF-like and p.R202Q carrier patients following LPS + UCN-01 treatment. Monocytes from HD (*n* = 12), FMF patients (*n* = 3), FMF-like patients (*n* = 5) and p.R202Q patients (*n* = 15) were treated with 12.5 µM UCN-01 after LPS priming (10 ng/mL) as described in Materials and Methods. p.R202Q patients were divided into homozygous (*n* = 7) and heterozygous (*n* = 8) groups for comparison. IL-1α levels were quantified by ELISA.**(a)** IL-1α levels after LPS + UCN-01 treatment, **(b)** IL-1α levels after LPS stimulation. Data are shown as the median (IQR). p calculated according to the Kruskal-Wallis test. Dunn’s post hoc test: *p < 0.05. The difference between homozygous vs. heterozygous groups was evaluated using the Mann Whitney test, ns. Abbreviations are as follows: FMF, Familial Mediterranean Fever; HD, healthy donors; homo, homozygous; het, heterozygous

It is reported that GSDMD, processed by caspase-1, forms plasma membrane pores that mediate Ca2 + influx, involved in the calpain-dependent maturation of IL-1α and in the release of this cytokine [22]. We evaluated IL-1α release from monocytes stimulated with LPS + UCN-01 among the groups (Fig. 5a, Figure S1c). We found no significant differences between the IL-1α levels released from p.R202Q monocytes and HDs. Instead, monocytes from FMF patients released higher levels of IL-1α than monocytes from HDs. No differences were observed in IL-1α release between monocytes from homo and heterozygous p.R202Q patients treated with LPS + UCN-01 (Fig. 5a). Interesting, although not significant, we observed a difference between HDs and p.R202Q patients in the release of IL-1α after monocytes LPS priming (Fig. 5b).

### Cytological and Cellular Morphology Evaluation of Leukocytes in FMF, FMF-like and p.R202Q Carrier Patients

**Fig. 6:**
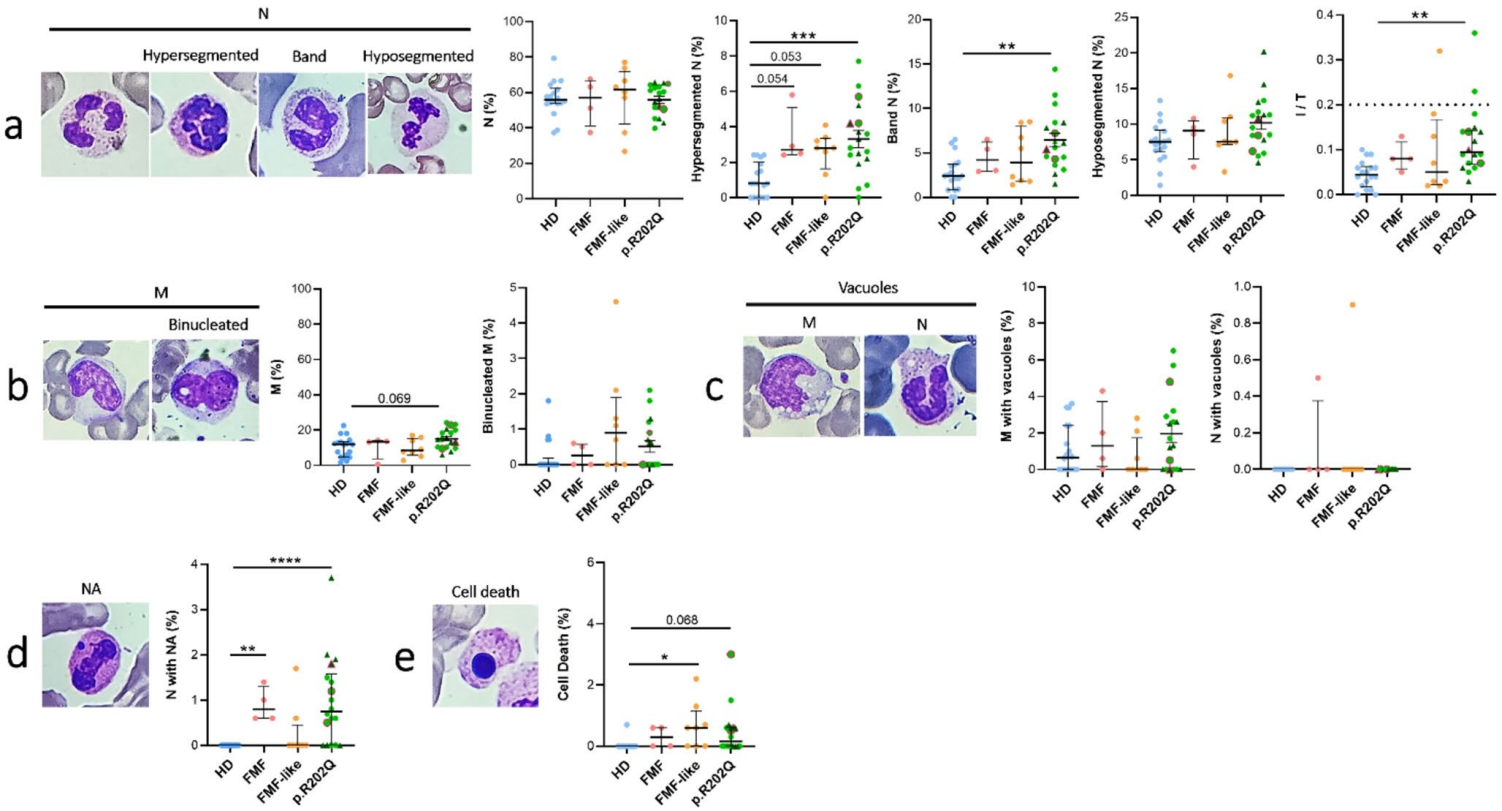
Cytogenic and cellular morphology evaluation of N and M from FMF, FMF-like and p.R202Q carrier patients. Peripheral blood smears from FMF (*n* = 4), FMF-like (*n* = 8), p.R202Q patients (*n* = 18) and HD (*n* = 18) were stained using MGG staining. **(a)** Percentage of N, hypersegmented, band, hyposegmented N and Immature N/ total N ratio (I/T index) **(b)** Percentage of M and binucleated M. **(c)** Percentage of M and N with vacuoles, **(d)** Percentage of N with NA, **(e)** Rate of cell death. Data are shown as the median (IQR). p calculated according to the Kruskal-Wallis test. Dunn’s post hoc test: *p < 0.05, **p < 0.01, ***p < 0.001, ****p < 0.0001. Abbreviations are as follows: FMF, Familial Mediterranean Fever; HD, healthy donors; N, neutrophilis; M, monocytes; NA, nuclear abnormalities

We evaluated the percentage of neutrophils (N), their stage of maturation, and cytological characteristics in all blood smears from patients and HDs. We found no difference in the percentage of neutrophils (N) between all groups analyzed. Hypersegmented N, cells that have remained in circulation for an extended period instead of migrating into the tissues, were higher in FMF, FMF-like and p.R202Q patients than in HDs (Fig. 6a). A left shift indicates the presence of immature N in blood and usually indicates an inflammatory leukogram. Band N were higher in p.R202Q patients than in HDs. A moderate shift to the left (0.2–0.29) and severe shift to the left (≥ 0.3) were observed in 2 p.R202Q patients and in 1 K695R het patient, and this index was higher in p.R202Q patients than in HDs. Finally, no differences were observed in the rate of M and binucleated M between all groups included in the study (Fig. 6b).

No differences were found in the percentage of M and N with vacuoles between al patients and HDs (Fig. 6c). The percentage of N with NA were higher in FMF patients and p.R202Q patients than in HDs (Fig. 6d). Finally, the rate of cell death was higher in FMF-like patients than HDs. Although not significant, we found a higher cell death rate in leukocytes from p.R202Q patients than HDs (*p* = 0.068) (Fig. 6e).

## Discussion

The association between mutations in the *MEFV* gene and FMF was reported for the first time in 1997. It has been shown that variants in exon 10 account for almost 80% of typical FMF cases. Genetic analysis may be performed to confirm FMF diagnosis since it increases the sensitivity of the criteria recently proposed by Eurofever/PRINTO [23]; however, the previous diagnostic criteria did not include genetic testing as part of the normal work-up [24, 25]. Indeed, there is a lack of conclusive genetic evidence for around 30% of patients diagnosed with clinical FMF [26].

This study focused on the p.R202Q gene variation, that is commonly considered as a polymorphism according to the Genome Aggregation Database (gnomAD); currently, there are limited data about the significance of this alteration, however, recent studies have shown that it may be a disease-causing mutation [1, 15]^27^– [30].

Therefore, this study aimed to investigate the role of *MEFV* p.R202Q alteration in a cohort of patients who received a diagnosis of probable FMF based on Tel Hashomer criteria. In these patients, we evaluated several aspects linked to inflammasome activation and inflammation: the patients’ clinical profile, the alteration of plasma cytokines, the response of monocytes / whole blood to PKN inhibition and finally, the cytological evaluation of leukocytes.

Our p.R202Q carriers indeed shared clinical similarities with canonical FMF, including a positive response to colchicine, with no differences between p.R202Q homozygous or heterozygous patients.

We observed a marked increase in IL-1β levels both in FMF and FMF-like patients, whereas p.R202Q patients exhibited lower levels; IL-18 levels were higher in FMF than in HDs, but no differences were found between p.R202Q patients and controls.

Proinflammatory IL-1β and IL-18 released by monocytes from HDs, FMF, FMF-like and p.R202Q patients were measured upon LPS + UCN0-1 treatment. Monocytes from FMF patients secreted significantly more IL-1β and IL-18 than HDs and p.R202Q monocytes after LPS + UCN-01 treatment. Three patients with the p.R202Q variant exhibited an increase in IL-1β that exceeded 600 pg/ml. These three patients underwent a repeat genetic analysis via Next-Generation Sequencing (NGS) panel, showing the presence of other gene variations, all recognized as VUS, on *IFIH1*, *TNFRSF1A* and *MVK* genes, respectively. We cannot exclude that these variants may have influenced both the clinical phenotype and the response to the LPS + UCN-01 functional assay. Statistical analyses performed after excluding these individuals from the p.R202Q patient group showed that the functional assay response in the remaining p.R202Q patients was comparable to that of HD (Figure S3 - S6). These findings may suggest that patients with p.R202Q, as well as other subjects who are likely to have other unrecognized or non-classically pathogenic variants, should be carefully evaluated through functional assays and genetic sequencing through panels with Next-generation Sequencing (NGS) approach or whole exome sequencing (WES) should be considered to avoid the risk of undertreatment.

The functional assay may also be performed using a small volume of human whole blood, supporting a more convenient and straightforward approach for FMF diagnosis. The preliminary data obtained with this assay are comparable to those from the monocyte-based assay; we did not observe differences in IL-1β and IL-18 release between p.R202Q patients and HDs. However, this assay requires further validation with a larger sample size as it may be affected by donor variability (leukocyte composition, disease activity at the time of sampling) and ongoing pharmacological treatments.

Inflammasome activation in FMF patients, mediated by PKC inhibition, was blocked by colchicine in line with the efficacy of this drug in patients. In p.R202Q patients, colchicine reduced the release of IL-18 and IL-1β only in the three individuals who exhibited inflammasome activation following LPS + UCN-01 treatment and who also carried mutations in the *IFIH1*, *MVK*, or *TNFRSF1A* genes (Figure S2). Notably, all three patients also showed a clinical response to colchicine in vivo. However, it is important to note that the in vitro response to colchicine observed in this functional assay does not fully reflect the patients’ clinical response to treatment. This assay specifically evaluates the effect of colchicine in the context of UCN-01–mediated pyrin inflammasome activation. In contrast, the clinical efficacy of colchicine in vivo likely results from its multiple pharmacological effects, including inhibition of neutrophil activation, modulation of chemokines and prostanoid production, as well as reduction of neutrophil and endothelial cell adhesion [31].

Another factor that suggests the activation of pyrin is the induction of pyroptosis. During inflammasome activation, GSDMD forms plasma membrane pores that facilitate Ca^2+^ influx, resulting in the calpain-dependent maturation of IL-1α and its release from pores [22]. In p.R202Q patients the levels of IL-1α released from UCN-01 treated monocytes were comparable to those found in HDs, suggesting that this variant does not alter pyrin function.

Several studies reported that the frequency of p.R202Q homozygosity is very low, and it has been associated with autoinflammatory manifestations, suggesting a potent dosage-dependent pro-inflammatory effect of this variant [29]. However, our data showed no differences between homozygous and heterozygous p.R202Q patients in the release of IL-1β, IL-18 and IL-1α from LPS + UCN-01 treated monocytes.

Taken together, these data (release of IL-1β, IL-18, IL-1α, and differences between homozygous and heterozygous patients) showed that the p.R202Q variant responded similarly to wild-type pyrin.

The impact of LPS, widely recognized as a potent activator of monocytes, in this study was useful to investigate the difference in the responsiveness of p.R202Q monocytes compared to HDs. An unexpected result was the response of p.R202Q monocytes to LPS treatment. We found that IL-1β release was higher in p.R202Q monocytes than in HDs, a mechanism probably not related to pyrin activation. Further studies will be necessary to assess whether the release of IL-1β after LPS treatment may depend on the activation of an “alternative inflammasome” pathway in p.R202Q patients.

Finally, we performed a cytologic evaluation of leukocytes from these patients to evaluate the presence of genomic instability, cell death rate, and abnormal neutrophilic subsets. Neutrophils are the most abundant circulating leukocytes during attacks in FMF patients and they rapidly change their characteristics as they get activated [32–35]. We found a high percentage of hypersegmented N and band N in FMF, FMF-like and p.R202Q patients. A heterogeneous population of these cells (hypersegmented and band N) might reflect their activated state even in the absence of acute attack. These data might suggest that patients experience chronic low-grade inflammation even between flares. Hypersegmentation of N was found in many microenvironments and disease states (cancer and acute - chronic inflammation) [36]. However, the functional consequences of this hypersegmented nuclear morphology are unknown and future studies are required to characterize this population and to evaluate their function. Finally, we found higher levels of cell death rate and NA in patients than HDs. This could be related to inflammation-induced cell death and nuclear damage. The study conducted by Varga et al. reported indeed that cell death mechanisms (e.g. NETosis and pyroptosis) can occur in neutrophils from inactive FMF patients [37]. Future studies will be needed to assess the role of pyroptosis in the pathogenesis of FMF.

In conclusion, our findings suggest that the p.R202Q variant does not interfere with the second regulatory mechanism of pyrin activation. Nevertheless, we acknowledge that this assay does not rule out the possibility that p.R202Q may impact pyrin function through alternative, as yet unidentified, mechanisms. Therefore, while our data do not support a direct functional consequence of p.R202Q in this context, the presence of this variant may still contribute to the clinical phenotype observed in some patients. From a diagnostic standpoint, the inclusion of p.R202Q in routine *MEFV* genetic screening may remain relevant, particularly in complex cases (potential involvement of additional genes responsible for autoinflammatory conditions) or when present in combination with other *MEFV* variants.

We would be remiss not to mention some of the limitations of our study. Firstly, the p.R202Q patients’ recruitment: we did not evaluate the possible carriage of *MEFV* p.R202Q mutation in the control group; rather, we requested the genetic test after choosing individuals who displayed symptoms typically of FMF, even though it is a common variation in the population. However, despite selecting patients with autoinflammatory symptoms, p.R202Q patients showed an almost absent response to UCN-01 stimulation compared to FMF patients. Given the potential classification of p.R202Q as a low-penetrance variant, a distinct functional response would be expected at least in homozygous individuals. However, in our study, we did not observe any significant differences in inflammasome activation or cytokine release between homozygous and heterozygous p.R202Q carriers. Moreover, previous data from a cohort of 218 healthy donors recruited in our geographical area showed that the percentage of individuals homozygous for the p.R202Q variant was very low (0.07%) [38]. Based on this, we performed an additional analysis (Figure S7), comparing the response to the LPS + UCN-01 treatment in homozygous p.R202Q patients with HDs. No significant differences were observed in the functional assay response, suggesting that the presence of the p.R202Q variant in the homozygous state does not result in abnormal pyrin activation. Although sequencing HDs would have provided another layer of certainty, our overall data indicate that the p.R202Q variant does not appear to impair the secondary regulatory mechanism of pyrin.

Secondly, our sample size was too small to draw any definitive conclusions. A comparison involving a larger cohort of FMF patients, stratified by genotype and including other VUS and benign variants, should be performed to better define the functional relevance of these findings.

Thirdly, the inclusion of patients who were receiving colchicine treatment at the time of sample collection. While we acknowledge that colchicine could potentially influence certain cellular responses, we believe that it does not significantly impact the final results of our functional assays. In particular, for the functional assay performed on isolated monocytes, the cells were cultured in colchicine-free conditions for 24 h, allowing for a washout of the drug. This approach minimizes the influence of colchicine treatment on the assay outcome. Furthermore, previous studies that used this type of assay have shown that colchicine did not affect the final outcome of the assay [6]. Colchicine treatment could potentially influence neutrophil counts, however, in our cohort, we found no significant alterations in this parameter. Studies in the literature suggest that colchicine primarily affects neutrophil function rather than morphology, and the usual colchicine doses do not result in significant cytological abnormalities [32, 39, 40].

## Electronic Supplementary Material

Below is the link to the electronic supplementary material.

Supplementary Material 1

## Data Availability

Data is provided within the manuscript or supplementary information files.
