# Supplementary material for: Unresolved Issues in Familial Mediterranean Fever: Is p.R202Q MEFV Variant Potentially Pathogenetic in Unleashing Inflammation?
Source: J Clin Immunol. 2025 Jun 10;45(1):104. doi: 10.1007/s10875-025-01898-8 (PMC12152063; doi:10.1007/s10875-025-01898-8)
Supplement: Supplementary file 1 — Supplementary Material 1 [file 10875_2025_1898_MOESM1_ESM.docx]

**Supplementary**

**Tables**

**Table S1.** SAA and CRP levels in FMF, FMF-like and p.R202Q patients

|  | **Patient Code** | **SAA** | **CRP** |
| --- | --- | --- | --- |
| **FMF** | 1  2  3  4 | negative  negative  negative  12.8 mg/L | negative  negative  negative  negative |
| **FMF-like** | 1  2  3  4  5  6  7  8 | 55.0 mg/L  negative  negative  negative  negative  negative  negative  50.0 mg/L | 6.0 mg/L  negative  negative  negative  negative  negative  negative  negative |
| **p.R202Q** | 1  2  3  4  5  6  7  8  9  10  11  12  13  14  15  16  17  18 | negative  negative  negative  negative  negative  negative  negative  negative  negative  negative  14.0 mg/L  16.4 mg/L  negative  negative  negative  negative  12.9 mg/L  negative | negative  negative  negative  negative  negative  negative  negative  negative  negative  negative  negative  negative  negative  negative  negative  negative  negative  negative |

Abbreviations are as follows: FMF, Familial Mediterranean fever; CRP, C-reactive protein; SAA, serum amyloid A

**Figures**


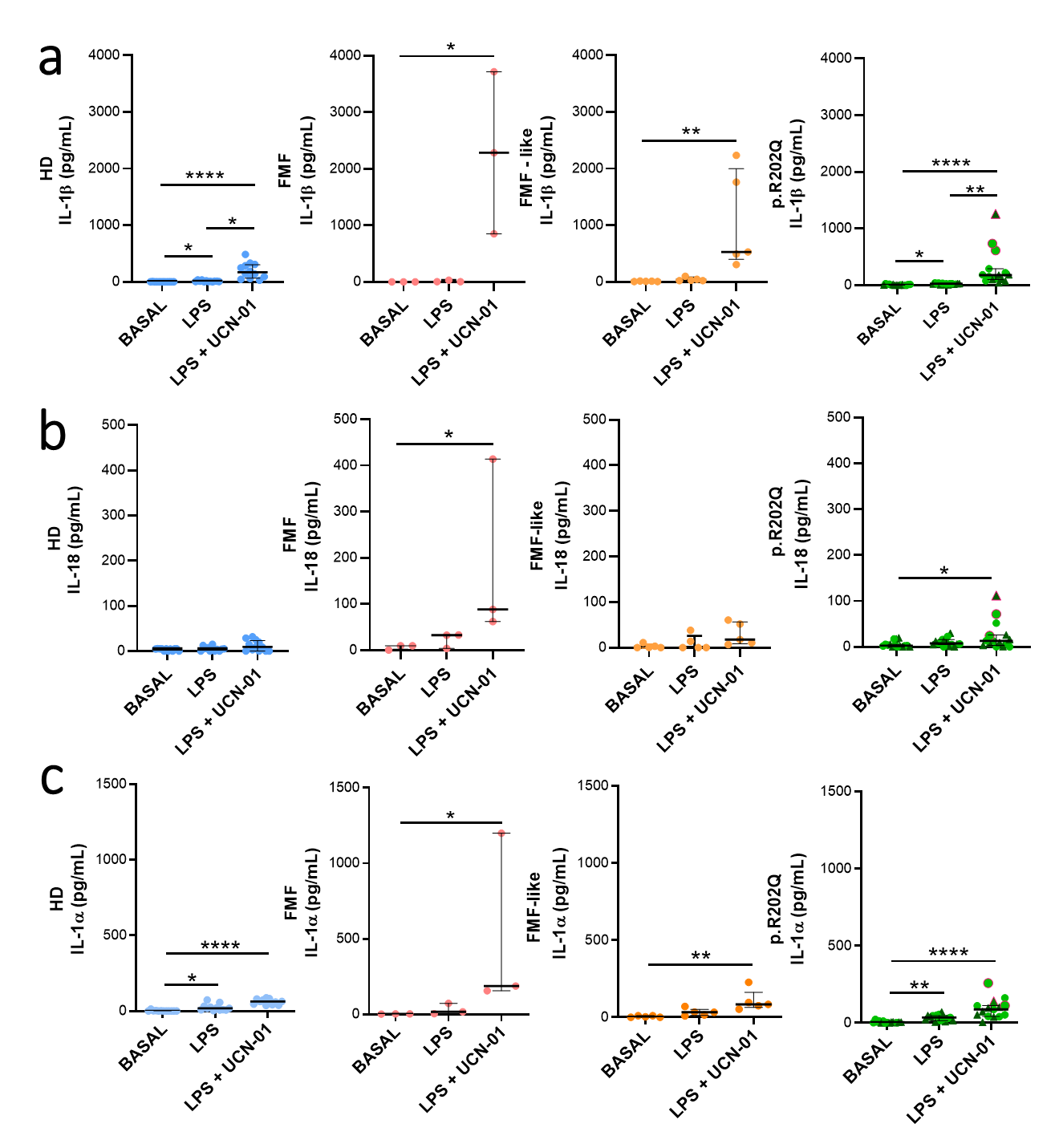


**Figure S1.** Evaluation of IL-1β, IL-18 and IL-1α levels in monocytes from HDs, FMF, FMF-like and p.R202Q carrier patients following LPS + UCN-01 treatment. Monocytes from HD (n = 12), FMF patients (n = 3), FMF-like patients (n = 5) and p.R202Q patients (n = 15) were treated with 12.5 µM UCN-01 after LPS priming (10 ng/mL) as described in Materials and Methods. Cytokine levels were quantified by ELISA. (a) IL-1β levels (b) IL-18 levels (c) IL-1α levels. Data are shown as the median (IQR). p calculated according to the Kruskal-Wallis test. Dunn’s post hoc test: *p<0.05, **p<0.01, ****p<0.0001. Abbreviations are as follows: FMF, Familial Mediterranean Fever; HD, healthy donors; homo, homozygous; het, heterozygous.


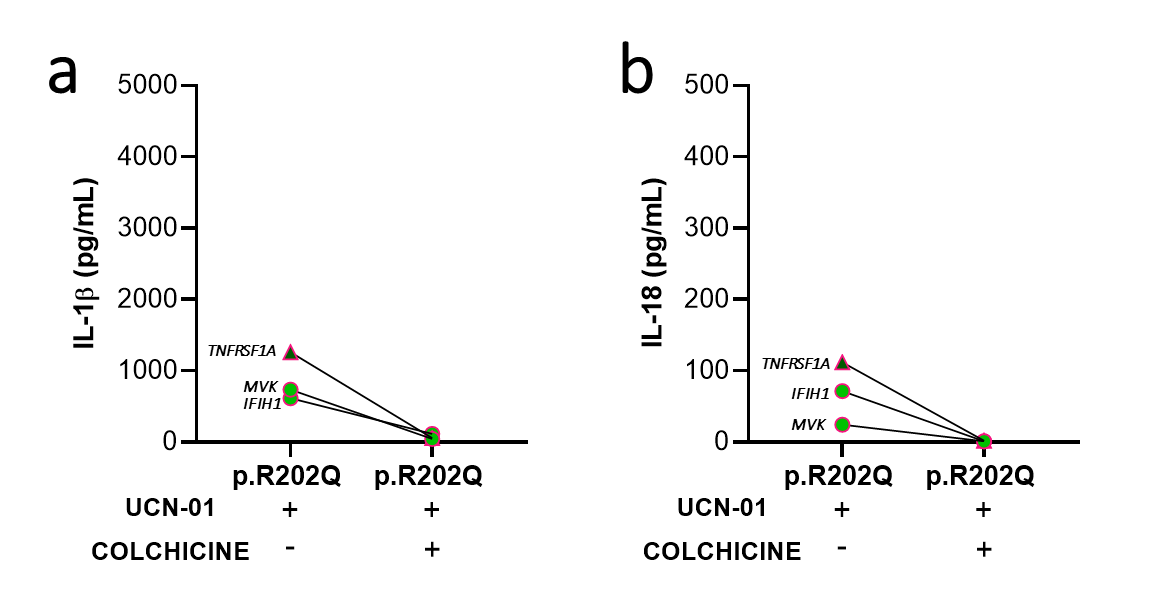


**Figure S2.** Colchicine blocked inflammasome activation only in monocytes from three p.R202Q patients following LPS + UCN-01 treatment. Monocytes from patients (n = 3) carrying the p.R202Q variant in the *MEFV* gene and p.V5F in the MVK gene, p.R186C in the *IFIH1* gene and p.R92Q in the *TNFRSF1A* gene were treated with 12.5 µM UCN-01 after LPS priming (10 ng/mL). Colchicine (1 µM) was added 30 min before addition of 12.5 µM UCN-01 as described in Materials and Methods. IL-1β and IL-18 levels were quantified by ELISA. (a) IL-1β levels after colchicine treatment. (b) IL-18 levels after colchicine treatment.


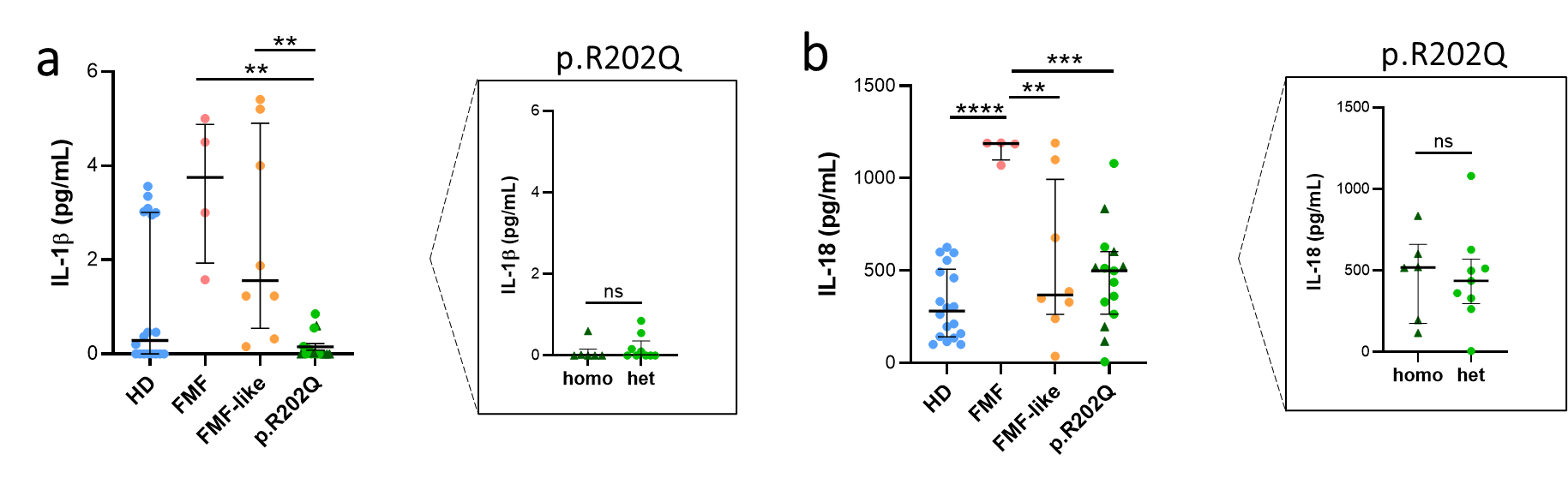


**Figure** **S3**. Plasma cytokines levels in FMF, FMF-like, p.R202Q patients and HD. Plasma cytokines levels in HD (n = 18), FMF (n = 4), FMF-like (n = 8) and p.R202Q patients (n = 15) were evaluated by ELISA as described in Materials and Methods. p.R202Q patients were divided into homozygous (n = 7) and heterozygous (n = 11) groups for comparison. (a) IL-1β levels. (b) IL-18 levels. Data are expressed as the median and IQR. p calculated according to Kruskal Wallis test. Dunn’s post hoc test: *p<0.05, **p<0.01, ***p<0.001, ****p<0.0001. The difference between homozygous vs heterozygous groups was evaluated using the Mann Whitney test, ns. Abbreviations are as follows: FMF, Familial Mediterranean Fever; HD, healthy donors; homo, homozygous; het, heterozygous.


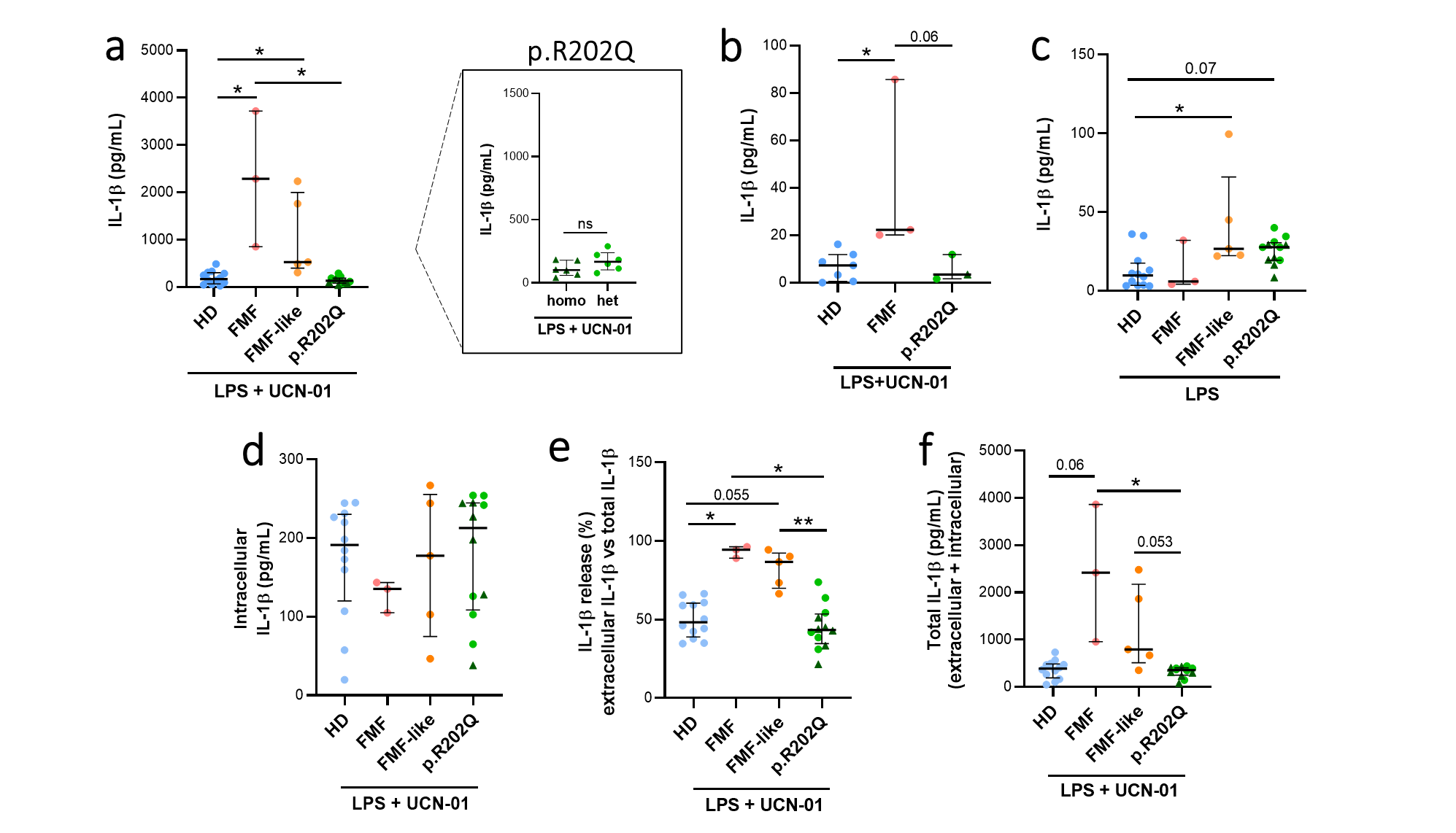


**Figure** **S4**. Evaluation of IL-1β levels in monocytes and whole blood from FMF, FMF-like and p.R202Q carrier patients following LPS + UCN-01 treatment. Monocytes from HD (n = 12), FMF patients (n = 3), FMF-like patients (n = 5) and p.R202Q patients (n = 12) and whole blood from HD (n = 7), FMF patients (n = 3) and p.R202Q patients (n = 3) were treated with 12.5 µM UCN-01 after LPS priming (10 ng/mL) as described in Materials and Methods. p.R202Q patients were divided into homozygous (n = 7) and heterozygous (n = 8) groups for comparison. IL-1β levels were quantified by ELISA. (a) Extracellular IL-1β levels in monocytes after LPS + UCN-01 treatment. (b) IL-1β levels after whole blood stimulation with LPS + UCN-01. (c) Extracellular IL-1β levels in monocytes after LPS stimulation. (d) Intracellular IL-1β levels (pro IL-1 β). (e) % of IL-1β release. (f) Total IL-1β levels (extracellular + intracellular IL-1β). Data are shown as the median (IQR). p calculated according to the Kruskal-Wallis test. Dunn’s post hoc test: *p<0.05, **p<0.01. The difference between homozygous vs heterozygous groups was evaluated using the Mann Whitney test, ns. Abbreviations are as follows: FMF, Familial Mediterranean Fever; HD, healthy donors; homo, homozygous; het, heterozygous.


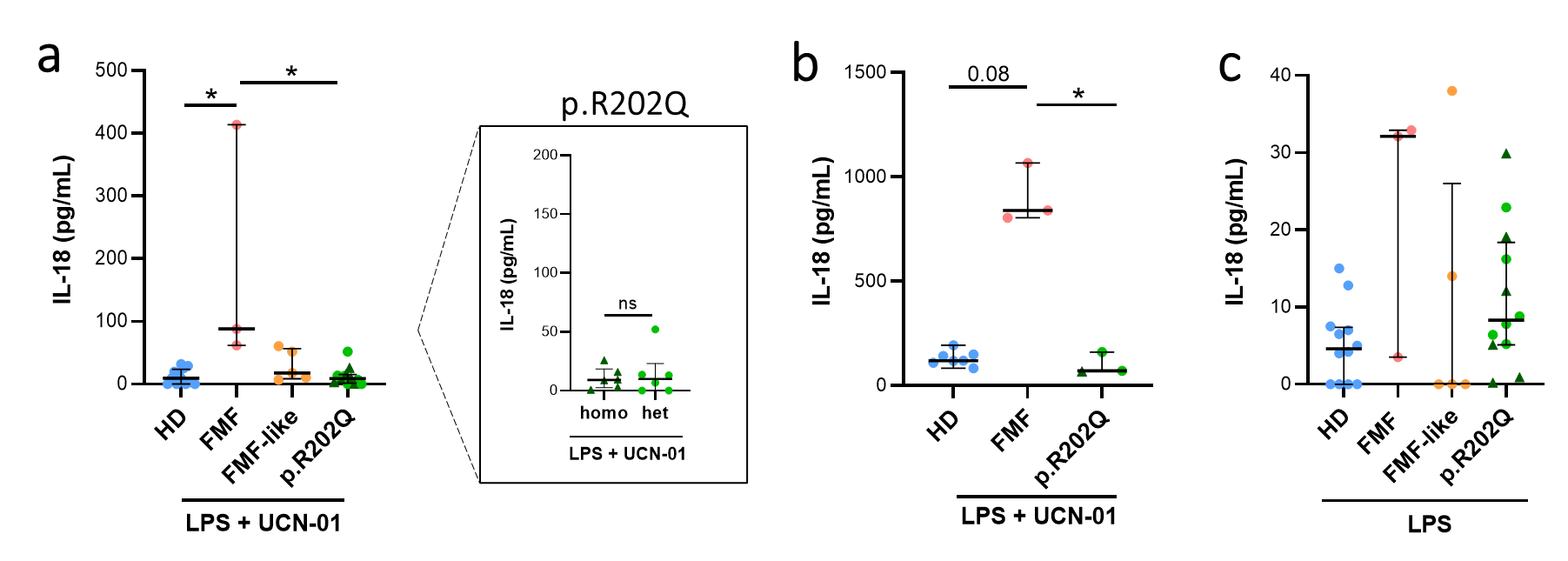


**Figure** **S5**. Evaluation of IL-18 levels in monocytes and whole blood from FMF, FMF-like and p.R202Q carrier patients following LPS + UCN-01 treatment. Monocytes from HD (n = 12), FMF patients (n = 3), FMF-like patients (n = 5) and p.R202Q patients (n = 12) and whole blood from HD (n = 7), FMF patients (n = 3) and p.R202Q patients (n = 3) were treated with 12.5 µM UCN-01 after LPS priming (10 ng/mL) as described in Materials and Methods. p.R202Q patients were divided into homozygous (n = 7) and heterozygous (n = 8) groups for comparison. IL-18 levels were quantified by ELISA. (a) IL-18 levels in monocytes after LPS + UCN-01 treatment. (b) IL-18 levels after whole blood stimulation with LPS + UCN-01. (c) IL-18 levels in monocytes after LPS stimulation. Data are shown as the median (IQR). p calculated according to the Kruskal-Wallis test. Dunn’s post hoc test: *p<0.05. The difference between homozygous vs heterozygous groups was evaluated using the Mann Whitney test, ns. Abbreviations are as follows: FMF, Familial Mediterranean Fever; HD, healthy donors; homo, homozygous; het, heterozygous.


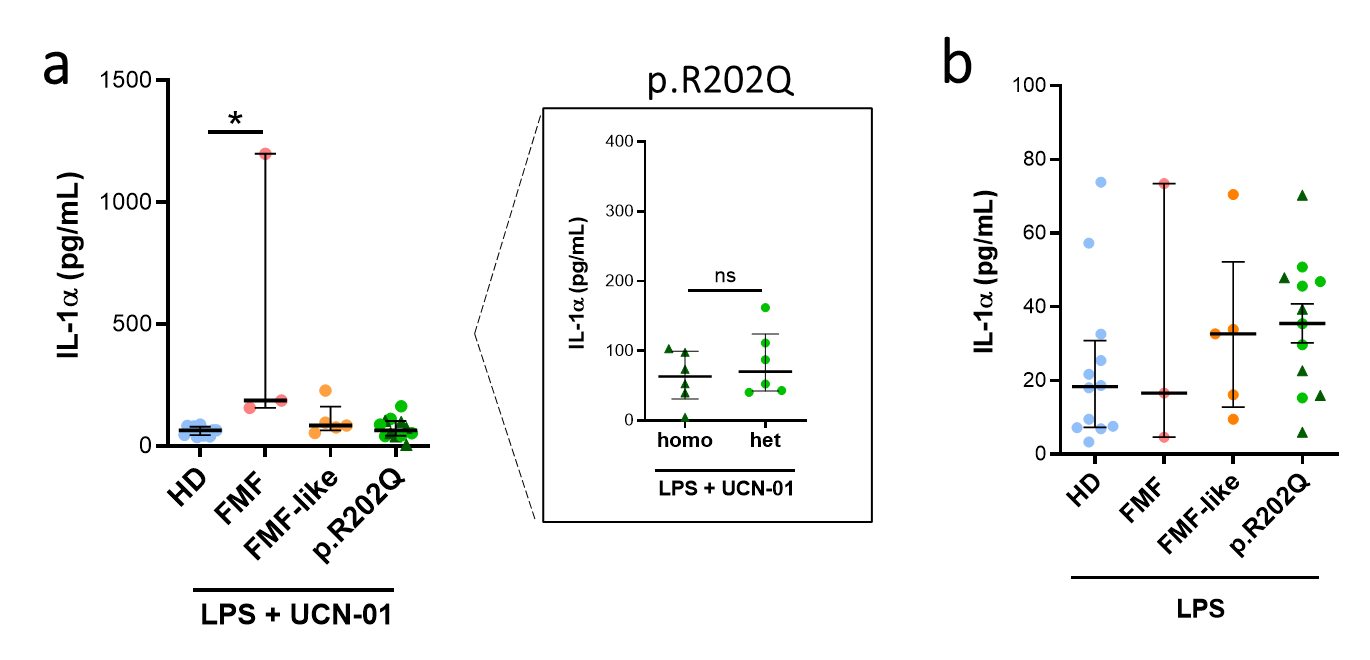


**Figure** **S6**. IL-1α release in monocytes from FMF, FMF-like and p.R202Q carrier patients following LPS + UCN-01 treatment. Monocytes from HD (n = 12), FMF patients (n = 3), FMF-like patients (n = 5) and p.R202Q patients (n = 12) were treated with 12.5 µM UCN-01 after LPS priming (10 ng/mL) as described in Materials and Methods. p.R202Q patients were divided into homozygous (n = 7) and heterozygous (n = 8) groups for comparison. IL-1α levels were quantified by ELISA. (a) IL-1α levels after LPS+UCN-01 treatment, (b) IL-1α levels after LPS stimulation. Data are shown as the median (IQR). p calculated according to the Kruskal-Wallis test. Dunn’s post hoc test: *p<0.05. The difference between homozygous vs heterozygous groups was evaluated using the Mann Whitney test, ns. Abbreviations are as follows: FMF, Familial Mediterranean Fever; HD, healthy donors; homo, homozygous; het, heterozygous.


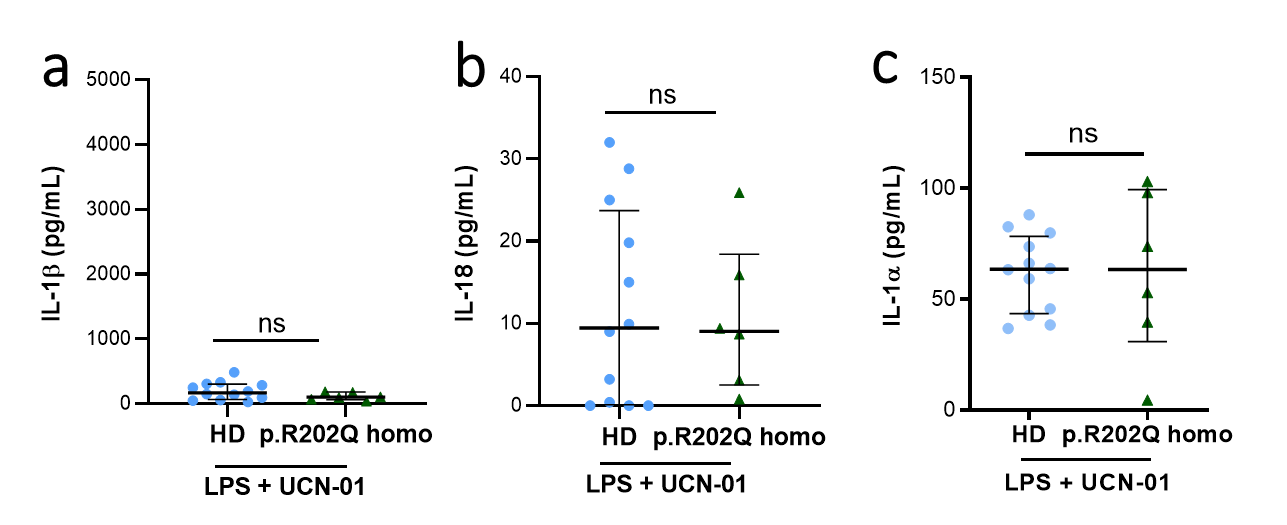


**Figure S7**. Evaluation of IL-1β, IL-18 and IL-1α levels in monocytes from HD and homozygous p.R202Q carrier patients following LPS + UCN-01 treatment. Monocytes from HD (n = 12) and homo p.R202Q patients (n = 6) were treated with 12.5 µM UCN-01 after LPS priming (10 ng/mL) as described in Materials and Methods. IL-1β, IL-18 and IL-1α levels were quantified by ELISA. (a) IL-1β levels (b) IL-18 levels (c) IL-1α levels. Data are shown as the median (IQR). p calculated according to the Mann Whitney test, ns. Abbreviations are as follows: HD, healthy donors; homo, homozygous.
